# A smartphone application to reduce problematic drinking: a feasibility trial

**DOI:** 10.1186/s40814-023-01420-0

**Published:** 2024-02-20

**Authors:** Christian Aljoscha Lukas, Jens Blechert, Matthias Berking

**Affiliations:** 1 Friedrich-Alexander-University Erlangen-Nuremberg, Naegelsbachstr 25a, 91052 Erlangen, Germany; 2 Department of Psychology, Centre for Cognitive Neuroscience, Paris-Lodron-University of Salzburg, Hellbrunner Str. 34, 5020 Salzburg, Austria

**Keywords:** Problematic drinking, Alcohol, Smartphone-based intervention, Mobile health, Brief intervention

## Abstract

**Background:**

Problematic drinking is common among college students and associated with various somatic and mental health problems. Given significant evidence for the efficacy of smartphone-based interventions and the frequent use of smartphones among college students, it can be assumed that such interventions have great potential to facilitate access to evidence-based interventions for students suffering from problematic drinking. Thus, we developed a brief intervention that combined a counseling session with an app that utilizes approach-avoidance modification training to reduce alcohol consumption.

**Methods:**

To test the feasibility and explore the potential efficacy of the intervention, we conducted a before-after single-arm study with *N* = 11 participants reportedly engaging in problematic drinking, who were instructed to practice with the app for 14 days. Feasibility was assessed with the System Usability Scale (SUS). Outcomes included the reduction of self-reported problematic drinking behavior, dysfunctional attitudes about alcohol, and craving, as well as implicit associations between alcohol and self during the training period. Additionally, self-reported problematic drinking behavior was assessed at a 4-week follow-up.

**Results:**

On average, participants rated app usability on the SUS (possible range: 0 to 100) with *M* = 84.32 (*SD* = 6.53). With regard to efficacy, participants reported a significant reduction of problematic drinking behavior (*d*_*pre vs. post*_ = 0.91) which was sustained at follow-up (*d*_*follow-up vs. baseline*_ = 1.07). Additionally, participants reported a significant reduction of dysfunctional attitudes about alcohol (*d*_*pre vs. post*_ = 1.48). Results revealed no significant changes in craving nor in implicit associations regarding alcohol.

**Conclusions:**

Findings from this feasibility study provide preliminary evidence that smartphone-based interventions might help reduce problematic drinking in college students. Further research needs to replicate these findings with larger samples in randomized controlled trials.

**Trial registration:**

DRKS00014675 (retrospectively registered).

## Key messages regarding feasibility

1. What uncertainties existed regarding the feasibility?
  Major uncertainties included (a) usability of the prototype app targeting problematic drinking, (b) possible effects of the intervention on dysfunctional attitudes towards alcohol and craving, (c) possible effects on implicit associations between alcohol and self during the training period, and (d) possible effects on problematic drinking behavior.

2) What are the key feasibility findings?
  The rating of usability was high, problematic drinking behavior was reduced, and dysfunctional attitudes towards alcohol were reduced. No significant changes in craving nor in implicit associations regarding alcohol were found.

3) What are the implications of the feasibility findings for the design of the main study?
  The findings of this feasibility study indicate that the intervention under investigation holds the potential for the reduction of problematic drinking behavior, as well as for the reduction of dysfunctional attitudes towards alcohol. Further development of the intervention is needed to improve the effect on craving and on implicit associations regarding alcohol.

## Background

Problematic drinking comprises heavy or hazardous consumption of alcohol and is a common experience in college students [1]. Studies on alcohol misuse indicate that up to 37% of the US college students regularly engage in binge drinking [2] and that two in five college students meet the criteria for problematic drinking [3]. Problematic drinking has been shown to be associated with a broad range of negative health, legal, and social consequences such as unwanted sexual contacts, unintended physical injuries, assaults, death, arrests, fines, and relationship difficulties [4, 5]. Moreover, it has been linked to several serious mental disorders including social anxiety disorder [6, 7], depression [8], posttraumatic stress disorder [9], and attention-deficit/hyperactivity disorder [10].

Because of the large number of students suffering from problematic drinking, there is a great demand for cost-effective interventions that are easy to disseminate [11]. Hence, in the past decade, various brief and often online-based interventions for problematic drinking in college students have been developed and evaluated [12–14]. Findings from these studies provide evidence for the efficacy of these interventions. However, averaged effect sizes ranging from *g* = 0.23 to 0.36 [13] show that there is still significant room for improvement.

Because of the widespread use of smartphones and their constant availability, research has recently focused on smartphone-based treatments for problematic drinking [15, 16]. Preliminary evidence for the efficacy of these treatments includes a study in which a 6-week smartphone-based skills training was shown to be superior to a waitlist control condition with regard to reducing excessive alcohol consumption in college students reporting excessive use of alcohol [17]. However, several other studies found no significant effects for smartphone-based interventions and commonly report comparatively low compliance and high drop-out rates [18, 19]. Findings showing that compliance is strongly associated with outcome [20] support the hypothesis that including measures that aim to enhance compliance—such as combining brief face-to-face counseling sessions with a smartphone-based skill training—might help to fully exploit the potential of smartphone-based interventions.

Another potentially effective way of improving the efficacy of smartphone-based interventions for problematic drinking might be the inclusion of approach-avoidance modification of trainings (AAMTs [21]). Such trainings usually present pictures of stimuli likely to cue or inhibit the tendency to consume alcohol (e.g., pictures of alcoholic beverages vs. non-alcohol beverages). Participants are then instructed to push alcohol-related pictures away from themselves and pull abstinence-related pictures towards themselves. Traditionally, AAMTs are computer-based and use a joystick to move stimuli away or towards oneself. Typically, they utilize a zoom feature that enlarges pictures when they are drawn towards oneself and diminishes them when they are pushed away from oneself.

Evidence for the efficacy of AAMT in the treatment of alcohol-related disorders includes a study in which Wiers and colleagues [22] found four sessions of AAMT as an adjunct to 3 months of CBT-based inpatient treatment for alcohol dependence to reduce relapse rate during the 1-year follow-up period by 13%. These findings were replicated by Eberl and colleagues [23]. With regard to problematic drinking, evidence is yet inconsistent. In a study conducted by Wiers and colleagues [24], one session of AAMT was found to significantly reduce self-reported drinking in hazardous drinkers. However, two other studies matching this procedure [25] failed to replicate these findings.

In an attempt to further improve the availability and efficacy of brief interventions for problematic drinking in college students, we developed a new intervention (mentalis Appstinence; MT-APS) that combines a brief face-to-face counseling session with 14 days of app-based AAMT. For the latter, we implemented an app in which alcohol-associated and abstinence-associated stimuli are presented on the smartphone screen and in which “swipe”-movements are used to move (and thereby enlarge/diminish) stimuli away from or towards oneself. Stimuli include both pictures of alcoholic and non-alcoholic beverages as well as attitudes that are likely to either prompt the use of alcohol or interfere with such use.

Preliminary evidence for the efficacy of combining AAMTs with brief face-to-face interventions comes from studies showing that such an approach can be effective in reducing body dissatisfaction [26], procrastination [27], and alexithymia [28]. To explore whether this approach might also be effective for treating problematic drinking in college students, we adapted previously used counseling sessions as well as previously used AAMT stimuli to problematic drinking in college students.

The present study was conducted in order to test the feasibility of MT-APS. Specifically, we aimed to assess the acceptance of the intervention in a sample of college students (objective 1) and to test whether MT-APS has the potential to reduce self-reported problematic drinking behavior (objective 2), dysfunctional attitudes towards alcohol (objective 3), alcohol-related craving (objective 4), and the strength of implicit associations between self and alcohol (objective 5).

## Methods

### Participants and procedures

Participating university students were recruited through flyers distributed on the campus of Friedrich-Alexander-University Erlangen-Nuremberg and in general practitioners’ offices, as well as through ads in social media channels and university websites. In all flyers and ads, we asked whether potential participants engaged in problematic drinking behavior and, if this was the case, offered the opportunity to participate in a study testing the feasibility and potential efficacy of a new psychological intervention against problematic drinking. In an attempt to minimize self-selection effects, the flyer did not provide more detail on the nature of the intervention.

Students interested in participating were asked to complete an online questionnaire screening for inclusion criteria. Potential participants had to meet the following criteria to be included in the study: (a) problematic alcohol use as indicated by a score ≥ 8 on the Alcohol Use Disorders Identification Test (AUDIT [29]), (b) access to a smartphone using Android version 4.0 or above, (c) age ≥ 18, (d) sufficient German language skills, and (e) informed consent. To maximize external validity, no exclusion criteria were applied. We assessed 42 potential participants for eligibility. Out of these 42 potential participants, nine individuals did not meet the inclusion criteria as they scored below eight in the AUDIT. The remaining 33 participants were contacted by email and provided with written information about the study procedures. From this subsample, 21 individuals did not respond to the contact email and one individual failed to provide informed consent. Data of the remaining 11 individuals participating in the study were assessed before and after the 14-day treatment period. Although a small sample, the size is in line with the recommendation by Julious [30]. With the exception of the strength of alcohol-related attitudes and cravings, baseline assessments were scheduled prior to the counseling session. As alcohol-related attitudes and pictures cuing craving were personalized in the counseling session, the strength of these attitudes and cravings were assessed after the counseling session and before the app-based training. In addition to pre- and post-treatment assessment, we assessed problematic drinking behavior at a 4-week follow-up.

**Fig. 1:**
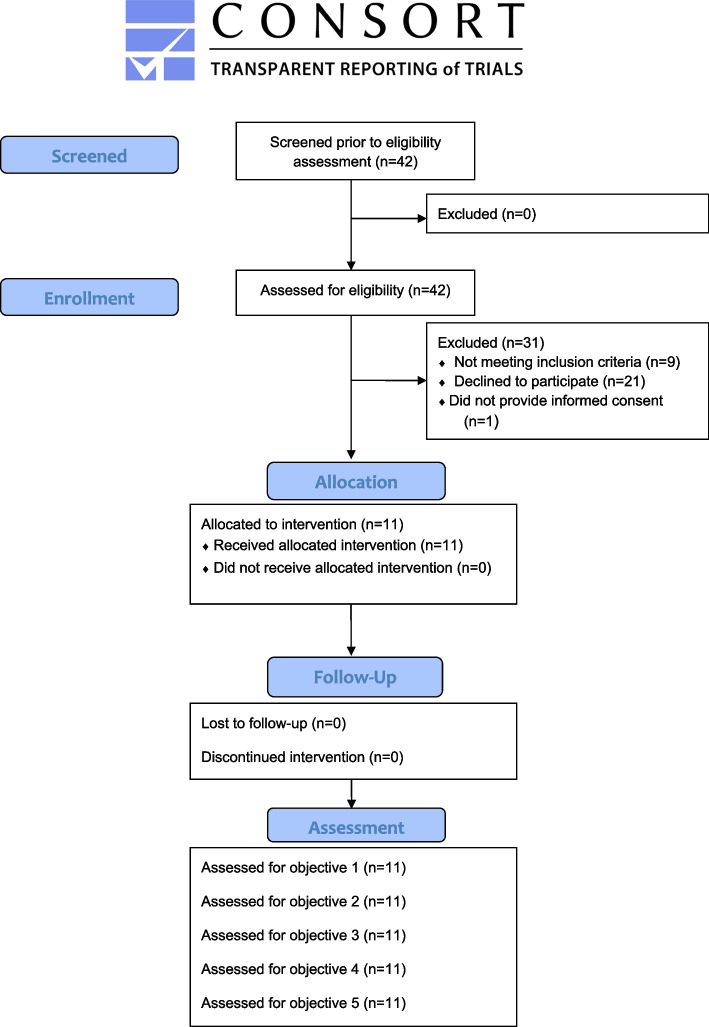
CONSORT flow diagram

Baseline and post-assessment took place in the department’s outpatient clinic. The 4-week follow-up was assessed with an online assessment tool (www.soscisurvey.de). Participant flow is illustrated in Fig. 1. All study procedures complied with the human research guidelines of the Helsinki Protocol and were approved by the ethics committee of the German Psychological Society (MB 072016_rev).

### Intervention

#### Intervention development

The protocol for the intervention and the app content was developed by the first and the senior author. As part of the development of the intervention, a preliminary version of the protocol (with 3 instead of 14 days of app-based training) was completed by a focus group comprising five master’s students in psychology who subsequently provided feedback on participant load and usability. The feedback was used to develop the final protocol for the intervention.

#### Brief counseling

In the counseling session (45 min), we first provided participants with general information about problematic drinking including possible negative consequences of hazardous alcohol use. Utilizing techniques from motivational interviewing [31], we then worked to enhance participants’ motivation to reduce problematic drinking by inquiring about personally relevant, negative consequences (e.g., “Why do you want to change your drinking behavior?”). In a third step, we educated participants about attitudes likely contributing to problematic drinking, assessed personally relevant dysfunctional attitudes, and informed participants that the AAMT included in MT-APS aims to reduce the salience of these attitudes. Finally, we assessed whether participants would rather tend to drink in positive or in negative mood states.

Counseling was delivered by two master’s students in psychology who were trained in the treatment protocol prior to study commencement. This training was provided jointly by the first (graduate psychologist in advanced clinical training) and last author (licensed psychologist and supervisor, professor in clinical psychology). It consisted of three 45-min sessions. The first session included a rehearsal of the nosology, the etiology of problematic drinking, and of evidence-based treatments for problematic drinking—with a particular focus on cognitive-behavioral therapy, AAMTs, and motivational interviewing. In the second session, the counselors reviewed attitudes likely to promote or interfere with problematic drinking and practiced to introduce participants to the idea that their attitudes would affect their drinking behavior. Subsequently, they were introduced to the sequence of steps they were to address in the counseling session (as described above). In the third session, students role-played the counseling and received feedback from the first author on how they could improve their performance. To further ensure treatment fidelity, counselors met with the first author after each counseling session and discussed how to overcome potential challenges to adherence to the protocol.

#### Mood induction

Based on evidence that mood is an important antecedent of alcohol use (e.g., [32]) and that, in some individuals, problematic alcohol consumption is cued by positive mood [33], whereas, in others, it is cued by negative mood [34], we reasoned that an AAMT in which participants practice avoidance towards alcohol-related stimuli and approach towards abstinence-related stimuli would be particularly effective if the training was conducted in the particular mood state constituting a high-risk situation for the particular participant. Thus, after the counseling session, we asked whether participants rather tended to engage in problematic drinking in response to negative or in response to positive moods. Participants reporting problematic drinking as a response to negative mood were invited to complete a negative mood induction prior to each AAMT training session. Negative mood was induced by having participants listening to Tomaso Giovanni Albinoni’s *Adagio* in G minor with 50% of the original velocity while reading ten negative self-statements (e.g., “I am a loser”). Participants reporting problematic drinking as a response to positive mood were invited to complete a positive mood induction prior to each AAMT training session. Positive mood was induced by having participants listening to Wolfgang Amadeus Mozart’s *A Little Night Music* while reading ten positive self-statements (e.g., “I am feeling very good today”). The complete list of statements can be obtained in the supplemental material section. Evidence for the efficacy of these procedures includes studies showing that both the aforementioned music as well as the Velten statements [35] and in particular the combination of both are effective in inducing positive/negative mood [36].

#### MT-APS App

The 1.0 version of the MT-APS app used in the present study randomly displayed pictures and attitudes related to problematic drinking on participants’ smartphone screens. The stimuli set in each app included 80 stimuli related to problematic drinking (40 attitudes and 40 pictures), out of which 40 stimuli promoted alcohol use and 40 stimuli promoted abstinence. To increase the personal relevance of these stimuli, participants were asked to phrase 20 dysfunctional attitudes (e.g., “Drinking is the only way to make new friends”) and 20 functional attitudes (e.g., “I enjoy my night out without getting drunk”) that were of particular importance for themselves. To facilitate this task, participants were provided a pool with attitudes related to problematic drinking to choose from or use as examples. Attitudes in this pool were adapted from the Situational Confidence Questionnaire [37]. Additionally, each participant had to create a set of 20 pictures representing his or her alcohol use (e.g., alcohol beverage) and a set of 20 pictures best representing abstinence (e.g., non-alcohol beverage). For this task, participants were provided with pictures created for this study by the first and the senior author. After the individual set of attitudes and pictures was completed, participants were asked to rate the extent of agreement with the chosen attitudes and the intensity of craving cued by the pictures representing alcohol use or abstinence, respectively.

Stimuli first appeared small and then became larger until they filled almost the entire smartphone screen. Participants were asked to swipe stimuli promoting abstinence towards themselves and stimuli cuing alcohol use away from themselves.

Pictures and attitudes disappeared from the screen after participants had reacted by pulling or wiping. MT-APS provided either positive (smiling emoticon and the word “Correct!”) or negative (frowning emoticon, the word “Wrong!”, and a short vibration of the smartphone) feedback to each reaction. In addition to the immediate feedback, participants gained and collected a star for every five correct answers given.

Participants were instructed to practice with MT-APS (i.e., complete the mood induction and then swipe dysfunctional stimuli away from self and functional stimuli towards themselves) once per day for at least 5 min for the next 14 days (“After the mood induction, the app will show you either pictures of alcohol drinks or attitudes promoting drinking or of pictures displaying non-alcohol drinks and attitudes promoting abstinence. Please swipe pictures of alcoholic drinks and attitudes promoting drinking *away* from yourself and swipe pictures of non-alcohol drinks and attitudes promoting abstinence *towards* yourself.”). Subsequently, MT-APS was installed on participants’ smartphones. To foster adherence to this training protocol, email reminders were sent to participants on the 3rd, 7th, 10th, and 13th training days.

### Measures

#### Screening

The Alcohol Use Disorders Identification Test (AUDIT; [29]) is a 10-item screening inventory that assesses the amount of alcohol consumption, problematic drinking behavior, and problems related to alcohol use. For the cutoff used for the AUDIT in this study (≥ 8), a specificity of ≥ 90% for detecting hazardous alcohol use has been reported [38]. For the internal consistency, a Cronbach’s alpha of 0.85 has been reported [39]. In the current study, the internal consistency was *α* = 0.62.

#### Feasibility

*Usability* was assessed using the System Usability Scale (SUS [40]). The SUS is a widely used, standardized 10-item self-report measure that assesses the usability of a system (e.g., software, websites, etc.). Items (e.g., “I found the system easy to use.”) are rated on a Likert scale ranging from 0 (“strongly disagree”) to 4 (“strongly agree”). Scores for each question are converted, added together, and then multiplied by 2.5 to generate the total score which ranges from 0 to 100. Based on extensive research with the SUS, a total score of 68 is considered above average [40]. Previous studies on the internal validity of the scale indicate Cronbach’s alphas as high as 0.91 [41]. In the current study, the internal consistency of the SUS was *α* = 0.62. In an attempt to assess aspects of utility not included in the SUS, we also asked participants to rate two additional items (“I feel better since I started training with the app” and “I would recommend the app to a friend”) on a Likert scale ranging from 0 (“strongly disagree”) to 4 (“strongly agree”). In addition, participants were invited to provide short written feedback about the app.

#### Preliminary evaluation of efficacy

*Problematic drinking behavior* was assessed with an adapted version of the Trier Alcoholism Inventory (TAI [42]). The TAI is a 90-item scale assessing antecedents and consequences of excessive alcohol consumption on seven domains over the last 4 weeks. To reduce participants’ burden, we selected 21 items of the TAI directly related to problematic drinking behavior (e.g., “Did you have physical complaints after a night of heavy drinking?”). The items selected for this study can be found in the supplementary material. In previous studies, the 90-item version of the complete TAI showed internal consistencies ranging between *α* = 0.70 and *α* = 0.80 in a large German-speaking sample [42]. In the current study, the internal consistency of the focused 21-item version of the TAI was *α* = 0.73.

For the assessment of *dysfunctional alcohol-specific attitudes*, participants were asked to rate the extent of which they agreed with each of the 20 individual dysfunctional attitudes. Ratings ranged from 1 (“very”) to 5 (“not at all”) on a 5-item scale.

To assess *alcohol-related craving*, participants were asked to rate the 20 self-chosen pictures showing alcoholic beverages. To this end, we asked participants to rate their drinking urge (“How bad do you want to drink this beverage?”) on a 5-item scale ranging from 1 (“very much”) to 5 (“not at all”).

*Implicit associations between alcohol and self* were assessed using the Implicit Association Test (IAT [43]). The IAT is a computer-based test that uses response times in a classification paradigm to assess implicit associations between target concepts and their attributes. Faster responses are expected when the target concept is more strongly associated with the attribute in participants’ neural representation of both entities. In this study, we used the Brief Drinking Identity IAT (BDI-IAT), a test that is well-researched in student populations and measures associations with the self and drinking: “drinking” + “me” (& “abstaining” + “not me”) versus “drinking” + “not me” (& “abstaining” + “me”). The BDI-IAT has been shown to be a strong and consistent predictor of alcohol consumption, problems related to alcohol, and craving of alcohol [44]. In previous studies, the BDI-IAT showed good test–retest reliability (*r* = 0.70) but a low internal consistency of *α* = 0.51. In the current study, the internal consistency was also low with *α* = 0.45.

### Feasibility outcomes

The following criteria were determined to evaluate the success of feasibility:

1. Participants rate usability higher than average (> 68).
2. Pre- and post-comparisons of the TAI scores indicate that self-reported problematic drinking behavior is significantly reduced during the intervention period.
3. Pre- and post-comparisons show a significant reduction of dysfunctional attitudes towards alcohol.
4. Pre- and post-comparisons show a significant reduction of craving.
5. Pre- and post-comparisons of the IAT show a significant reduction of implicit associations between self and alcohol.

### Statistical analyses

To summarize findings on adherence and usability, we computed mean scores and standard deviations. For self-reported problematic drinking behavior, agreement with dysfunctional attitudes and alcohol-related craving, we compared means of pre- vs. post-assessment (and in the case of problematic drinking also means of pre- vs. follow-up assessment) with a dependent *t* test. For implicit associations, D scores were calculated [43] and changes in mean D scores between pre- and post-assessment were tested with a dependent *t* test. As effect size measure we report Cohen’s *d.* According to commonly used conventions [45], we a priori defined *d* = 0.2/0.5/0.8 as small/moderate/large effects. All statistical analyses were performed with SPSS 23.

## Results

### Participants and preliminary analyses

The mean age of the final sample of 11 student participants was *M* = 24.36 (*SD* = 4.03). Participants were predominately male (7 of 11) which can be regarded as representative of problematic drinking (e.g., [46]). Two participants self-reported a recently diagnosed mental disorder (both major depression, with one of the two participants reporting a comorbid attention-deficit hyperactivity disorder). The participant reporting a diagnosis of major depression with comorbid attention-deficit hyperactivity disorder received psychopharmaceutical treatment while participating in the study. The mean AUDIT score assessed at screening was *M* = 14.64 (*SD* = 5.22). Table 1 shows additional participants’ characteristics. For dysfunctional attitudes and problematic drinking behavior, all assumptions of normality were met. Normality was not met for craving, hence we used a Wilcoxon signed-ranks test.

**Table 1:** Demographic variables

Variable	Intervention group (*n* = 11)
Age, *M* (*SD)*	24.36 (4.03)
Gender (male), *n* (%)	7
Occupation *n* (%) Student	11
Diagnosed mental disorder (yes) *n* (%)	2
Family member with alcohol addiction (yes) *n* (%)	3

### Adherence to the intervention

All participants completed the counseling sessions. On average, participants used the app on 10.73 days (*SD* = 1.90, range = 5–14), completed a mean of 11.45 training sessions (*SD* = 13.64, range = 5–18), and spent a total time of 39.56 min on the app-based training (*SD* = 14.36 min, range = 13.63–62.90).

### Feasibility evaluation

Usability ratings as assessed with the SUS were high with a score of *M* = 84.32 (*SD* = 6.53, range = 75.00–92.50). Results on the two app-specific items yielded a score of *M* = 2.27 (*SD* = 1.19, range = 0–4) for “I feel better since I started training with the app” and a score of *M* = 2.80 (*SD* = 1.23, range 0–4) for “I would recommend the app to a friend”. Analyses of the written feedback revealed that four participants perceived non-alcoholic pictures showing unhealthy beverages (e.g., energy drinks or soft drinks containing large amounts of sugar) as not helpful, three participants mentioned that a stable internet connection was required for training with MT-APS and that this was at times difficult to ensure, two participants reported that it was not possible to read incoming text messages while training with the app, and one participant reported that he had experienced defective line breaks when MT-APS displayed stimuli showing lengthy attitudes.

### Preliminary evaluation of efficacy

Pre- and post-comparisons of the TAI scores indicated that self-reported problematic drinking behavior was significantly reduced during the intervention period (pre-intervention vs. post-intervention: *t*(10) = 3.014, *p* = 0.013; *d* = 0.91). This effect was sustained at follow-up (pre-follow-up vs. follow-up: *t*(10) = 3.54, *p* = 0.005; *d* = 1.07). With regard to dysfunctional attitudes towards alcohol, findings indicated a significant pre- vs. post-reduction in agreement with dysfunctional attitudes (*t*(10) = 4.92), *p* = 0.001; *d* = 1.48). No significant pre- vs. post-changes were found for craving (*Z* = 1.34, *p* = 0.180) and implicit associations between self and alcohol *t*(10) = 0.18, *p* = 0.859). All findings regarding the efficacy of the intervention are shown in Table 2.

**Table 2:** Descriptive statistics for evaluation of potential efficacy

	t1 *M* (*SD*)	t2 *M* (*SD*)	t3 *M* (*SD*)
TAI	49.55 (7.80)	45.18 (10.19)	44.27 (8.88)
IAT (drink)	0.38 (0.11)	-2.67 (5.56)	
IAT (abstain)	-0.08 (0.02)	-0.02 (0.47)	
Agreement with dysfunctional statements	3.49 (0.86)	2.42 (0.48)	
Craving of alcoholic beverages	3.61 (0.77)	2.72 (0.64)	

*TAI* Trier Alcoholism Inventory, *IAT* Implicit Association Test

## Discussion

In this study, we aimed to explore whether a blended, smartphone-based intervention that combined a 45-min counseling session with 14 days of app-based AAMT might be a feasible intervention for problematic drinking in college students. Usability ratings and written feedback from the participants provide preliminary evidence for sufficient usability and acceptance of the intervention. Additionally, a significant reduction of self-reported problematic drinking behavior during the intervention period is in line with the assumption that the intervention might be effective in reducing problematic drinking. The finding that these changes are still significant at follow-up provides preliminary evidence that these effects might be sustained over time. With regard to the size of these effects, it is of note that they appear above what is commonly reported for desktop-based online interventions targeting problematic drinking (e.g., [47]; *d* = 0.42) and within the range of effects obtained in app-based interventions (e.g., [48]; *d* = 0.87–1.37). As such, the findings suggest that MT-APS might potentially be a feasible and efficacious intervention for people suffering from problematic drinking.

It is of note that we found no significant effect on the IAT. This finding might indicate that the intervention does not change problematic drinking by modifying early responses (as intended in many AAMTs) [49] but, potentially, rather by changing alcohol-related attitudes. This hypothesis would be consistent with MT-APS presenting not only stimuli of alcoholic and non-alcoholic beverages but also of alcohol-related attitudes. To the best of our knowledge, there are no studies in the domain of alcohol use utilizing AAMTs with attitudes as stimuli. Thus, future research should work to clarify to what extent and in which populations a stronger focus on attitudes (as opposed to beverages) might improve the efficacy of AAMTs. Moreover, baseline measures of attitudes about craving and alcohol were assessed after the counseling session. Thus, the comparison of pre-and-post only reflects the effect of the application, not the effect of the combined intervention of counseling and an app-based AAMT. In future studies, attitudes about craving and alcohol should therefore be assessed before the counseling sessions. However, before such far-reaching conclusions can be drawn, significant limitations of the present study have to be taken into account. First, the present sample was very small and likely to consist of highly motivated participants. Such participants can be assumed to greatly benefit from any kind of treatment or even to reduce problematic drinking on their own account [50]. Thus, future studies need to replicate present findings with larger and more representative samples. Second, because of the lack of a control condition, it is unclear whether effects reported with regard to problematic drinking behavior and alcohol-related attitudes are due to time or unspecific factors (e.g., demand characteristics, social support, habituation in the mood-induction task, or expectations). Thus, future research should evaluate interventions such as MT-APS with the help of randomized, controlled designs that employ waitlist or sham control groups or even compare MT-APS with the present gold standard of (online and face-to-face) treatment for problematic drinking. Third, preliminary evaluation of potential effects of the intervention lacks the inclusion of commonly used and empirically validated instruments. As such, the abbreviated version of the TAI used in the present study has not been empirically validated. Moreover, problematic drinking behavior was exclusively assessed with the help of self-report and might, therefore, be biased by factors such as selective memory, self-serving self-perception, and impression management [51]. Thus, future research should complement the use of validated self-report instruments with the use of clinical interviews (assessing consumed standard drinks) and/or behavioral and biological indicators of alcohol use. Fourth, the strength of agreement with alcohol-related attitudes was assessed after the counseling session. Thus, changes occurring immediately during the counseling session were not assessed by this measure. Therefore, future studies should individualize attitudes prior to the counseling session and include the assessment of agreement with these attitudes in the baseline assessment. Fifth, significant effects on alcohol-related attitudes and non-significant effects on craving and implicit associations between self and alcohol provide some preliminary ideas with regard to the mechanisms responsible for the potential effects of MT-APS. To further clarify these mechanisms, future studies should combine the use of a larger sample size and a randomized controlled design with the utilization of mediation analyses [52]or other statistical methods developed to clarify causal pathways [53]. Ideally, such efforts would be complemented by dismantling studies that systematically vary potential mechanisms of change (in this case the presence vs. absence of counseling session or AAMT, resp.) and relate changes in outcome to these variations [54].

In sum, findings from the present study provide some encouraging results suggesting that smartphone-based interventions that combine counseling with app-based AAMT might represent a feasible and efficacious intervention against problematic drinking, which has the potential to be accepted by college students. However, future research needs to evaluate interventions such as MT-APS with larger sample sizes, more powerful designs, stronger assessment, and statistical procedures before such conclusions can be drawn in earnest.

## Data Availability

The datasets used and analyzed during the current study are available from the corresponding author on reasonable request.
